# Evaluation of a Three-Fluid Nozzle Spraying Process for Facilitating Spray Drying of Hydrophilic Polymers for the Creation of Amorphous Solid Dispersions

**DOI:** 10.3390/pharmaceutics15112542

**Published:** 2023-10-27

**Authors:** Lena Karin Mueller, Laura Halstenberg, Nicole Di Gallo, Thomas Kipping

**Affiliations:** Merck Life Science KGaA, Frankfurter Straße 250, 64293 Darmstadt, Germany; laura.halstenberg@merckgroup.com (L.H.); nicole.di-gallo@merckgroup.com (N.D.G.); thomas.kipping@merckgroup.com (T.K.)

**Keywords:** polyvinyl alcohol, amorphous solid dispersion, spray drying, spray-dried dispersion, process development, sustainability

## Abstract

Amorphous solid dispersions (ASDs) enable formulations to improve the solubility of poorly soluble active pharmaceutical ingredients (APIs). The amorphous state is reached through the disruption of the crystalline lattice of an API resulting in an increased apparent solubility with faster disintegration. Nevertheless, this form is characterized by a high-energy state which is prone to re-crystallization. To ensure a stable ASD, excipients, e.g., polymers that form a matrix in which an API is dispersed, are used. The applicable polymer range is usually linked to their solubility in the respective solvent, therefore limiting the use of hydrophilic polymers. In this work, we show the applicability of the hydrophilic polymer, polyvinyl alcohol (PVA), in spray-dried solid dispersions. Using a three-fluid nozzle approach, this polymer can be used to generate ASDs with a targeted dissolution profile that is characterized by a prominent spring and desired parachute effect showing both supersaturation and crystallization inhibition. For this purpose, the polymer was tested in formulations containing the weakly basic drug, ketoconazole, and the acidic drug, indomethacin, both classified as Biopharmaceutics Classification System (BSC) class II drugs, as well as the weakly basic drug ritonavir classified as BCS IV. Furthermore, ritonavir was used to show the enhanced drug-loading capacity of PVA derived from the advantageous viscosity profile that makes the polymer an interesting candidate for spray drying applications.

## 1. Introduction

In recent years, the creation of amorphous solid dispersions (ASD) has emerged as a promising strategy to enhance both the solubility and bioavailability of low water-soluble drug substances. The creation of such formulations usually involves the dispersion of a drug substance within a polymer matrix at a molecular level resulting in a homogenous amorphous mixture [1].

As ASDs are inherently unstable and tend to crystallize over time, one of the key challenges is maintaining the amorphous state during storage and handling. To overcome this limitation, various techniques have been explored to stabilize ASDs after production.

Mainly three preparation methods are used to create ASDs: melting methods, solvent evaporation, and mechanical activation methods. Different preparation methods also result in varying thermal histories and the amount of mechanical stress introduced into the material [2].

Classical spray drying (SD) processes are often limited due to low solubility of either the drug substance or required excipients. Preparations of two-phase systems like suspensions of colloids are often applied to overcome this issue. Four different types of atomizer technologies are used for most of the industrial SD applications: rotary atomizers, pressure nozzles, two-fluid nozzles and ultrasonic atomizers [3].

Hydrophilic polymers usually exhibit low solubility in organic solvents which is a critical drawback for applications like SD, where in most cases, the polymer is dissolved in a common solvent along with the drug compound to create a uniform solution which is then atomized into fine droplets for drying.

Attempts to enable the use of hydrophilic polymers for SD applications usually involve the creation of complex solvent systems and dedicated, often rather time-consuming preparation methods to create a common solvent system between low-soluble drug compounds and hydrophilic polymers. The resulting solid dispersions in many cases still indicate signs of crystallinity [4].

The use of three-fluid nozzle atomizers has been described for the development of dry powder combination products of theophylline and salbutamol for pulmonary delivery [5].

First attempts for the creation of solid dispersions via three-fluid nozzle systems were performed by Kauppinen et al. by investigating mannitol as a hydrophilic carrier for diazepam; instead of using one common solvent, two different solvents are used to prepare two different solutions to dissolve the carrier and API in separate vessels [6]. They are then pumped separately through two different channels and are combined at the tip of the nozzle, where they are rapidly mixed and atomized using pressurized gas added via the third channel.

The aim of this study is to evaluate a novel amorphization approach using a Multi Liquid Kinetic Technology (AMOR–MLKT approach). This process can serve as an alternative preparation concept to classical SD, enabling the use of hydrophilic polymers in spray drying applications while maintaining rapid preparation times and utilizing simple solvent systems. Immediate mixing of multiple solutions at the nozzle, combined with rapid drying kinetics assure a homogenous and stable ASD.

Various established polymers were evaluated during the study. Focus was also laid on a recently developed grade of polyvinyl alcohol (PVA). This grade differentiates from existing polymers due to its adapted hydrolysis degree (82%). It is of interest if adapted molecular properties leading to improved amphiphilicity enable the application of PVA for spray-drying approaches.

## 2. Materials and Methods

### 2.1. Materials

Polyvinyl alcohol 3-82, (Parteck^®^ MXP 3-82, EMPROVE^®^ ESSENTIAL, Merck KGaA, Darmstadt, Germany), polyvinyl alcohol 4-88 (Parteck^®^ MXP 4-88 EMPROVE^®^ ESSENTIAL, Merck KGaA, Darmstadt, Germany), hydroxypropyl methylcellulose acetate succinate HPMC-AS (Affinisol™, Colorcon, Harleysville, PA, USA), polyvinylpyrrolidon PVP K30 (VWR Chemicals, Darmstadt, Germany), polyvinyl caprolactam polyvinyl acetate-polyethylene glycol grafted copolymer (Grafted copolymer, Soluplus^®^ BASF, Ludwigshafen am Rhein, Germany), indomethacin (IND, Sigma Aldrich, St. Louis, MS, USA), ketoconazole (KET, Piramal, Mumbai, India), ritonavir (RIT, Aurobindo Pharma Ltd., Hyderabad, India), Norvir^®^ (Abbott, Lake Bluff, IL, USA), simulated gastric fluid SGF (protocol by USP), fasted-state simulated intestinal fluid FaSSIF (Biorelevant, London, UK), methanol (Sigma Aldrich), acetone (Sigma Aldrich), ethanol (Sigma Aldrich), acetonitrile (Sigma Aldrich), Na_3_PO_4_*12 H_2_O (Sigma Aldrich), 0.1 M HCl (Sigma Aldrich), diisopropylamin (Sigma Aldrich), ammonium acetate (MilliporeSigma, Burlington, MA, USA).

### 2.2. Overview APIs

Within this work, three different APIs were chosen to investigate the enhancement of dissolution of the respective spray-dried dispersions vs. the crystalline raw material. To investigate the polymer’s versatility, these APIs cover a broad range of properties. Indomethacin (IND) is regarded a weak acid and classified as BSC class II; ketoconazole (KET) is a weak basic drug in class II of the BSC’ and ritonavir (RIT) is in class IV of the BSC with a weakly basic behavior (Table 1).

### 2.3. Methods

Spray drying

Equipment: SD was performed on a Buchi B295 equipped with an inert loop and high-performance cyclone (Buchi Labortechnik AG, Flawil, Switzerland).

Parameters: inlet temperature 90 °C, outlet temperature 50 °C (for IND); inlet temperature 100 °C, outlet temperature 60 °C (for KET, RIT) with N_2_ as inert drying gas, flow rate of 35 m^3^/h and aspirator at 100%; atomizing air flow rate 670 L/min (N2 55 mm). Nozzles used: two-fluid nozzle and three-fluid nozzle for the AMOR–MLKT approach. The solid content was kept at 10% *w*/*w* for the mixture of polymer and API.

Solid dispersions of IND, KET and RIT: polymers were dissolved in water; IND was dissolved in a mixture of water to acetone in a proportion of 3:7; KET was dissolved in methanol; RIT was dissolved in ethanol. The solutions were spray dried using a three-fluid nozzle. The inner feed forwarded the API solution, the outer feed forwarded the respective polymer solution. A syringe pump (PHD ULTRA^TM^ Syringe Pump, Harvard Apparatus, Holliston, MA, USA) was used to forward both solutions at 4 mL/min. 

Solid dispersions of HPMC-AS and the API: both components were dissolved in methanol and spray dried using a two-fluid nozzle. For the two-fluid nozzle approach, the integrated Buchi pump was used at 18% feed rate. 

The neat API, RIT, was dissolved in ethanol and spray dried using a two-fluid nozzle.

Viscosity

The apparent viscosity of the solutions was measured using a Haake Mars 60 Rheometer (Thermo Fisher, Karlsruhe, Germany). The measurement was performed with the following parameters: temperature, 20 °C; mode, controlled rate (CR); shear rate, *ẏ* = 100 1/s; and time, 1 min.

SEM

Scanning electron microscopy (SEM) was performed using a Zeiss Gemini 460: Field Emission Gun (FEG cathode), variable pressure low-vacuum system, magnification 8 x–2000 kx, accelerating voltage 0.02 kV–30 kV, detectors: in-lens detectors, low-voltage BSE detector, SE detector, low vacuum SE detector, STEM detector, and 5 segment BSE detector.

XRPD

X-ray powder diffraction was measured using a Miniflex 600 X-ray diffractometer (Rigaku, Tokyo, Japan) with CuKα radiation (λ = 1.54 A), reflector mode 3°–50° 2θ (deg), scan speed 10° 2θ (deg)min, and step size 0.02θ (deg). Acceleration voltage 45 kV and 15 mA current.

Dissolution

Equipment: dissolution was performed with a Sotax AT7 smart (Sotax AG, Lörrach, Germany) equipped with an ultraviolet visible (UV-VIS) Agilent 8453 spectrophotometer.

Dissolution of IND: 25 mg of IND is added to 900 mL of SGFsp, Apparatus 2, paddle, 75 rpm at 37 °C. The drug concentration was measured spectrophotometrically at 318 nm with a pathlength of 10 mm.

Dissolution of KET: to assess the parachute effect, a pH shift was performed, 500 mg of KET was added to 750 mL of 0.1 M HCl, Apparatus 2, paddle, 50 rpm at 37 °C. After 120 min, pH was changed from 1.2 to 6.8 via addition of 250 mL 0.2 M Na_3_PO_4_*12 H_2_O. Drug concentration was measured using reversed-phase high-performance liquid chromatography (RP-HPLC).

For FaSSIF, 400 mg of API was added to 500 mL FaSSIF, Apparatus 2, paddle, 50 rpm at 37 °C. The drug concentration was determined via RP-HPLC.

Dissolution of RIT: 20 mg of API was dissolved in 20 mL FaSSIF. Drug concentration was measured using RP-HPLC. For FaSSIF, 400 mg of RIT was added to 500 mL of FaSSIF, Apparatus 2, paddle, 75 rpm at 37 °C. The drug concentration was measured spectrophotometrically at 215 nm with a pathlength of 0.5 mm.

RP-HPLC

Equipment: for RP-HPLC, an Agilent 1260 Infinity HPLC system (Agilent, Santa Clara, CA, USA) equipped with an Agilent 1260 II variable wavelength detector with a LC-18 (Supelco 4.0 × 300 mm, 5 µm) column at 40 °C oven temperature was used.

RP-HPLC of KET: following the USP dissolution protocol for ketoconazole tablets with mobile phase, eluent A = 10 mL diisopropylamin in 5 l methanol, eluent B = 5 g/L ammonium acetate in deionized water; linear gradient at 70:30 ratio eluent A to eluent B. Injection volume 5 µL at 2 mL/min flow rate with detection via UV Vis at 225 nm.

RP-HPLC of RIT: following the USP dissolution protocol for ritonavir with mobile phase, eluent A = 4.2 g/L potassium phosphate monobasic in water, eluent B = acetonitrile with a linear gradient of 45:55 eluent A: eluent B. Injection volume 20 µL at 1 mL/min flow rate and UV Vis detection at 215 nm.

## 3. Results and Discussion

### 3.1. Two-Fluid Nozzle vs. Three-Fluid Nozzle

To successfully generate an ASD via SD using a polymeric matrix, the API and polymer need to be fully dissolved in the feed solution to mitigate phase separation and ensure a molecularly dispersed solid solution [15,16]. Using a two-fluid nozzle, only one liquid feed can be applied in the process as the second channel forwards the spraying gas. This design enables droplet generation at the tip of the nozzle where the liquid feed and spraying gas come into contact. Several polymers are available that can be readily dissolved conjointly with the API such as hydroxypropyl methylcellulose HPMC (soluble in ethanol, methanol, ethanol/dichloromethane), hydroxypropyl methylcellulose acetate succinate HPMC-AS (soluble in methanol, acetone, ethyl acetate), and Copovidone PVP-VA64 (soluble in acetone, dichloromethane) [15,16].

**Figure 1:**
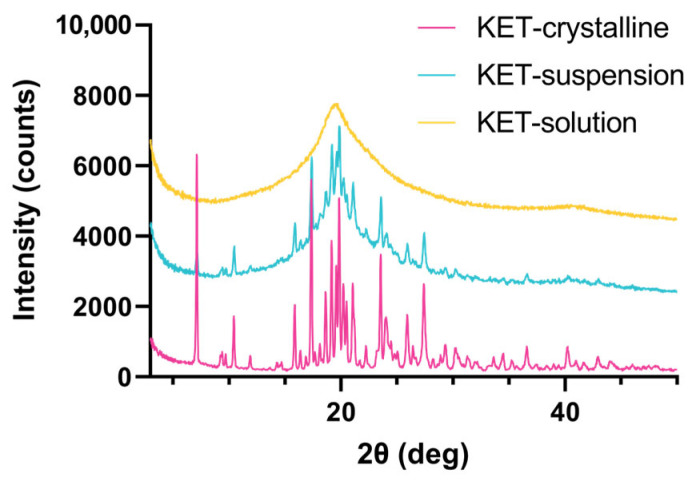
X-ray powder diffraction at reflector mode 3°–50° 2θ (deg), scan speed 10° 2θ (deg)min, step size 0.02θ (deg). Acceleration voltage 45 kV and 15 mA current. KET spray dried from a solution and suspension vs the crystalline substance. Solvent system 1:1 ethanol:water, PVA 4-88 as polymer. Solution: 14% DL KET, suspension: 30% DL KET.

Figure 1 illustrates how KET is not fully converted into the amorphous state when spray dried from a suspension. Peaks at 10.5, 17.2, 19.8, and 23.6 2θ (deg) can be allocated to the crystalline substance. Spraying from a solution yields an amorphous product displaying no peaks in the X-ray powder diffraction (XRPD). Here, PVA 4-88 was used as the hydrophilic carrier in a two-fluid nozzle set-up. For the API–polymer solution, a solvent system containing 1:1 ethanol:water was used to ensure both the solubility of the API and polymer and the applicability of this solvent system in the equipment. Only a 14% drug loading (DL) can be reached due to the API’s limited solubility in the aqueous solvent system. Increasing the DL leads to precipitation of the API, generating a suspension.

Circumventing the precipitation of the API and making sure the API and polymer are both fully dissolved in the respective solvent can be addressed by applying two individually tailored solvents to each component. With a three-fluid nozzle, one additional channel is available to forward a second solvent feed. The use of this three-fluid nozzle approach enables formulators to utilize the full portfolio of polymers, including those of hydrophilic nature that might not be applicable in a common solvent with the API. Using water as the solvent system for hydrophilic polymers and additionally dissolving only the API in organic solvent is a more sustainable approach in spray drying ASDs. Furthermore, the International Conference on Harmonization (ICH) advises the use of less toxic solvents in their Q3C (R8) guideline on impurities [17].

### 3.2. Evaluation of Viscosity

**Figure 2:**
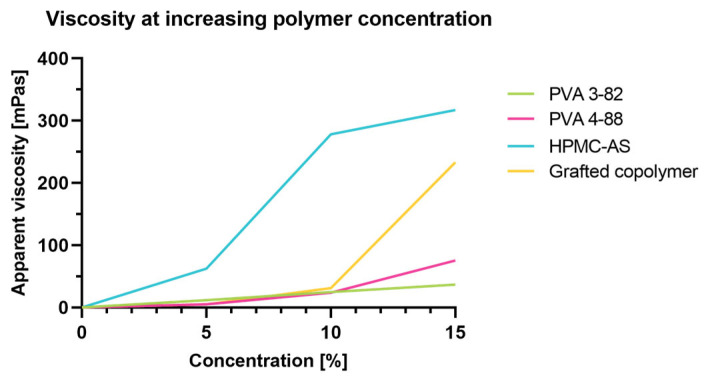
Apparent viscosity at increasing polymer concentrations.

To enable a homogenous process over the entire manufacturing time, the target viscosity is an important parameter. The low viscosity of PVA even at high solid content enables high loading during spraying. A direct comparison between chemically differing polymers is given in Figure 2. Cellulose-based polymers exhibit a concentration-dependent viscosity increase, whereas synthetic polymers usually provide lower viscosities at higher loadings. PVA especially shows advantages as it consists of linear polymer chains without larger side chain modifications.

**Figure 3:**
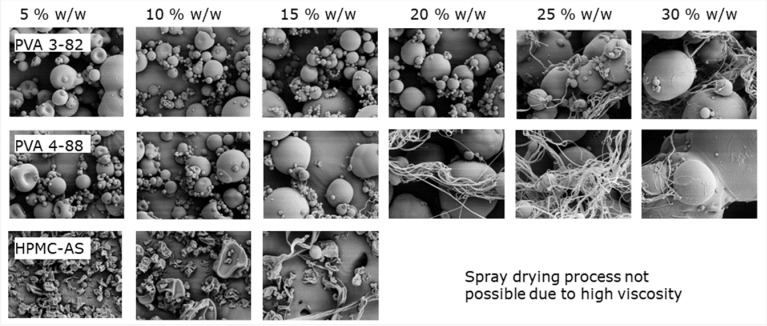
SEM images of spray-dried particles at different polymer concentrations.

Low viscosities enable a high loading of spraying solution. In Figure 3, a direct comparison of different PVA grades demonstrates a loading of up to 20% *w*/*w* with PVA 3-82, and up to 15% *w*/*w* with PVA 4-88, whereas for HPMC-based polymers, the solid loading is limited to 10% *w*/*w*. Processing these polymers at higher concentrations leads to clogging of the nozzle, making spray drying impossible. As seen in the scanning electron microscope (SEM) images, above the aforementioned concentrations, filaments start to become visible. During atomization, the feed exits the nozzle as a liquid sheet that subsequently forms a filament and then droplets. If the polymer solution gels before droplets can be formed, filament-like particles can be obtained. Besides negatively influencing the SD process, these particles have a further impact due to poor flowability and low density. Shephard et al. investigated filament formation using Eudragit L100, coming to the conclusion that with increasing polymer concentration in the liquid feed, formation of filaments became more prominent [18].

### 3.3. Spray Drying and Dissolution of Indomethacin 30% DL in SGF

Indomethacin is classified as a non-steroidal anti-inflammatory drug and reversibly inhibits the cyclooxygenase. Because of this, the generation of prostaglandins is decreased, resulting in an analgesic, anti-inflammatory and anti-pyretic effect [19]. It is defined as a BSC class II API, and as a weak acid, it shows limited solubility, especially in acidic media (Table 1). 

**Figure 4:**
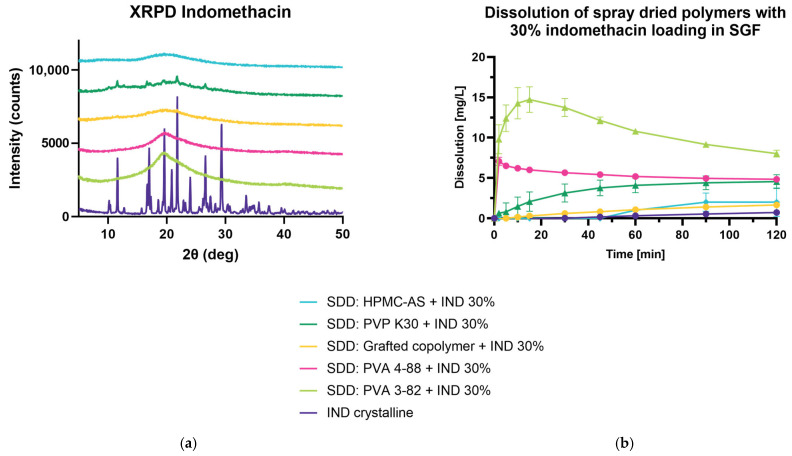
Spray-dried dispersion with 30% DL indomethacin. (**a**) X-ray powder diffraction at reflector mode 3°–50° 2θ (deg), scan speed 10° 2θ (deg)min, step size 0.02θ (deg). Acceleration voltage 45 kV and 15 mA current. (**b**) An amount of 25 mg of IND in 900 mL of SGFsp, Apparatus 2, paddle, 75 rpm at 37 °C, UV Vis detection at 318 nm.

Most in vitro dissolution studies are very limited and do not accurately reflect the environment which can be found in the gastrointestinal tract. Using biorelevant media, like fasted-state simulated intestinal fluid (FaSSIF) or simulated gastric fluid (SGF), which contain bile salts and phospholipids (for FaSSIF), or pepsin (SGF) can better predict in vivo behavior [7]. Therefore, solubility enhancement was investigated using SGF. Using the AMOR–MLKT approach, PVA 3-82, PVA 4-88, polyvinylpyrrolidone (PVP K30), and polyvinyl caprolactam polyvinyl acetate-polyethylene glycol (grafted co-polymer) were spray dried with IND to yield a spray-dried solid dispersion, HPMC-AS was spray dried using the two-fluid nozzle approach. The conversion to an amorphous state was determined by XRPD and showed that only the process using PVP K30 did not result in the desired product (Figure 4a). Peaks of the crystalline raw material were observed at 11.7, 17.1, 21.7, and 26.7 2θ (deg). Dissolution in SGF indicated that using PVA 3-82 leads to the desired dissolution profile, exhibiting a prominent spring within the first 10 min of measurement, and a steady decrease of dissolution showing a pronounced parachute behavior of the polymer (Figure 4b). The enhanced dissolution was followed by PVA 4-88, showing a fast onset and slow decrease but overall lower dissolution values. All other polymers showed a weaker dissolution enhancement of IND in SGF.

Shadambikar et al. investigated the release of IND from HME and vacuum compression molding (VCM) samples using PVA 4-88, showing a similar release profile in SGF. They found that this PVA grade was able to maintain a steady-state drug dissolution over 360 min [20].

### 3.4. ASD with Ritonavir

Ritonavir is a viral protease inhibitor used in the treatment of human immunodeficiency virus (HIV). The inhibition of the HIV aspartic protease leads to noninfectious and immature HIV particles and can be used in addition to reverse transcriptase inhibitors [21]. As ritonavir displays low solubility and low permeability, it is classified as a BSC class IV API. To investigate the dissolution enhancement of the spray-dried dispersions, dissolution measurements were conducted in FaSSIF.

#### 3.4.1. Spray Drying and Dissolution of Ritonavir at 30% DL in FaSSIF

**Figure 5:**
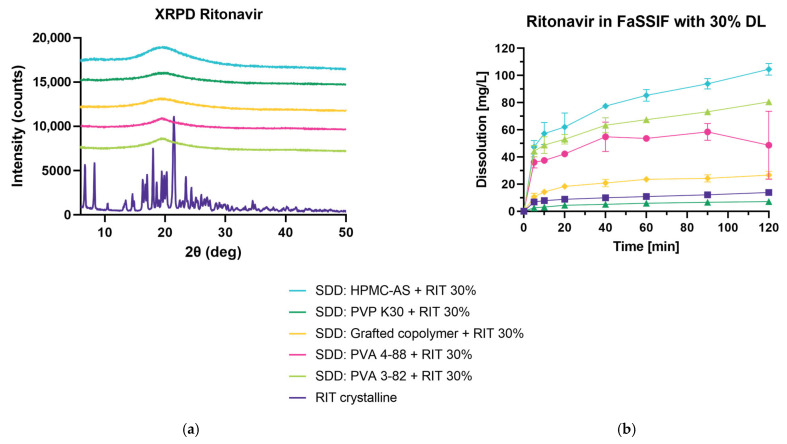
Spray-dried dispersion with 30% DL ritonavir. (**a**) X-ray powder diffraction at reflector mode 3°–50° 2θ (deg), scan speed 10° 2θ (deg)min, step size 0.02θ (deg). Acceleration voltage 45 kV and 15 mA current. (**b**) Mini dissolution of 1 mg/mL API in FaSSIF (20 mg RIT). Drug concentration is measured using RP-HPLC at 215 nm.

Figure 5a shows that all spray-dried dispersions show amorphization of the crystalline drug through the SD process using the AMOR–MLKT approach for PVA 3-82, PVA 4-88, PVP K30, and the grafted co-polymer, as well as the two-fluid nozzle set-up for HPMC-AS. Due to the high viscosity of HPMC-AS, the applicability in SD processes is limited (Figure 2). This can be seen when looking at the process yield which was calculated from the material readily available in the collection vessel. While the process with HPMC-AS results in a <1% yield, PVA 3-82 shows a >50% yield (Table 2). To optimize the process, the concentration of solid content during HPMC-AS spray drying should be reduced which would lead to an unfavorable increase of solvent.

All polymers, except PVP K30, show dissolution enhancement of RIT in FaSSIF (Figure 5b). Within the first 5 min, PVA 3-82, PVA 4-88, and HPMC-AS exhibit a prominent spring phase which shows the supersaturation of RIT in the dissolution media followed by a steady increase of release over the time of measurement. The spray-dried dispersion of HPMC-AS results in the highest dissolution values, followed by PVA 3-82 and PVA 4-88. Interestingly, the ASD containing PVP K30 was determined to be amorphous given the XRPD data, but in the dissolution experiment, it performed unexpectedly. Chan et al. performed dissolution experiments with ASDs containing ketoprofen and PVP K30 vs. PVPVA and PVA in which they demonstrated PVP K30 showing the slowest dissolution rate [22]. In their work, they concluded that even though completely amorphous, the PVP K30 sample did not result in enhanced dissolution due to hydrophobic agglomeration and re-crystallization hindering re-dissolution. Furthermore, Trasi et al. investigated 20% DL ritonavir in PVP showing that the PVP dispersion was not able to reach the amorphous solubility, whereas the sample prepared with HPMC-AS dissolved rapidly, reaching higher concentrations [23].

#### 3.4.2. Spray Drying and Dissolution of Ritonavir at Increasing DLs in FaSSIF

**Figure 6:**
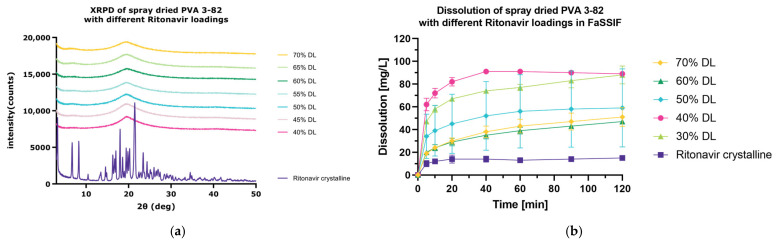
Spray-dried dispersion with PVA 3-82 and different drug loadings of ritonavir. (**a**) X-ray powder diffraction at reflector mode 3°–50° 2θ (deg), scan speed 10° 2θ (deg)min, step size 0.02θ (deg). Acceleration voltage 45 kV and 15 mA current. (**b**) Mini dissolution of 1 mg/mL API in FaSSIF (20 mg RIT). Drug concentration is measured using RP-HPLC at 215 nm.

As ritonavir is readily converted to the amorphous state, a drug loading escalation study was performed to evaluate the optimized drug loading using PVA 3-82 and RIT (Figure 6a). Therefore, different drug loadings (**40%**, 45%, **50%**, 55%, **60%**, 65%, **70%** *w*/*w*) of RIT within the polymer matrix were spray dried using the SD protocol and the DLs in bold were investigated regarding their dissolution enhancement behavior in FaSSIF vs. the crystalline drug and the 30% DL. All analyzed DLs show an enhanced dissolution compared to the crystalline drug (Figure 6b). It can be highlighted that a 40% *w*/*w* drug loading results in the highest dissolution enhancement, followed by the 30% DL. For each individual API in an ASD, the interplay between active and excipient determines the dissolution behavior and “sweet spot” in drug loading. It has been seen that, with rather low drug loadings, dissolution is controlled by the polymer, whereas at high drug loading, dissolution is API-controlled [24]. A congruent release of the drug results in higher dissolution values, whereas there is a switch to incongruent release which hinders dissolution. Here, we were able to see that the switch to incongruent release can be observed with the addition of 50% *w*/*w* API or more as the release of ritonavir in FaSSIF is not further increased. This emphasizes how PVA 3-82 can be used for achieving high drug loadings due to its optimized viscosity.

#### 3.4.3. Spray Drying and Dissolution of Ritonavir at 70% DL in FaSSIF

**Figure 7:**
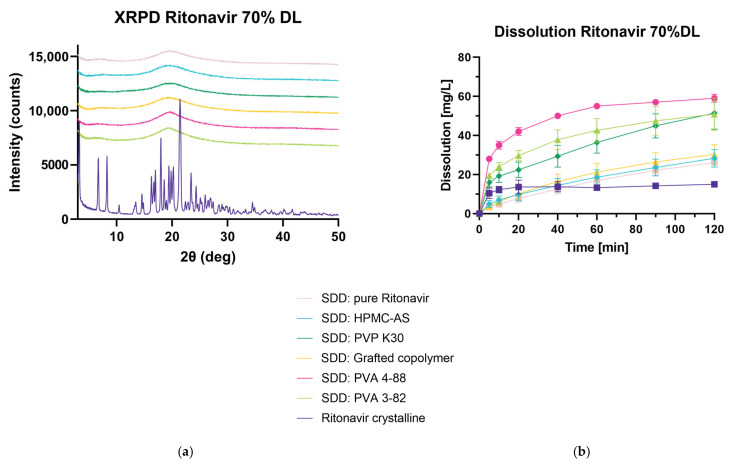
Spray-dried dispersion with 70% DL ritonavir. (**a**) X-ray powder diffraction at reflector mode 3°–50° 2θ (deg), scan speed 10° 2θ (deg)min, step size 0.02θ (deg). Acceleration voltage 45 kV and 15 mA current. (**b**) Mini dissolution of 1 mg/mL API in FaSSIF (20 mg RIT). Drug concentration is measured using RP-HPLC at 215 nm.

In a next step, 70% *w*/*w* DL of ritonavir was investigated using all polymers. Additionally, the raw material was spray dried, yielding an amorphous product (Figure 7a). All ASDs containing 70% DL were investigated in FaSSIF regarding their dissolution behavior (Figure 7b). At the final timepoint of 120 min, all spray-dried dispersions, as well as the spray-dried RIT, showed an enhanced release of RIT. PVA 4-88, followed by PVA 3-82 and PVP K30, showed superior behavior, while HPMC-AS and the grafted copolymer are comparable to the spray-dried raw material.

#### 3.4.4. Comparison of PVA 3-82-Ritonavir ASD with Marketed Formulation

**Figure 8:**
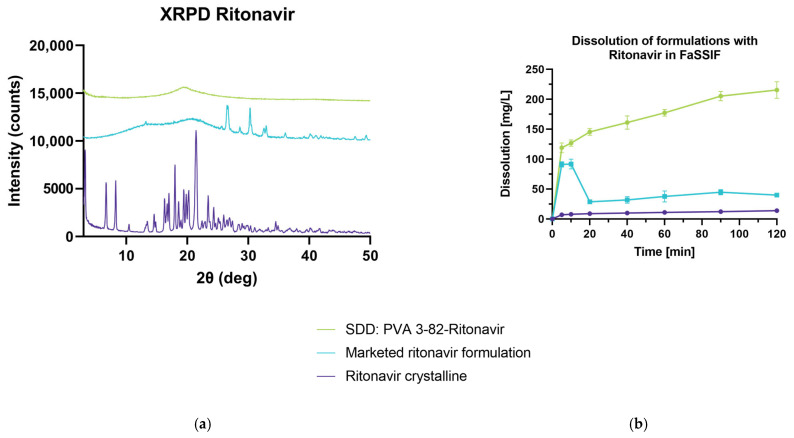
Spray-dried dispersion of PVA 3-82 with ritonavir vs. marketed formulation. (**a**) X-ray powder diffraction at reflector mode 3°–50° 2θ (deg), scan speed 10° 2θ (deg)min, step size 0.02θ (deg). Acceleration voltage 45 kV and 15 mA current. (**b**) An amount of 100 mg of RIT (calculated from DL) is added to 500 mL of FaSSIF, Apparatus 2, paddle, 75 rpm at 37 °C, UV Vis determination at 215 nm.

These results indicated that polyvinyl alcohol is a great fit for the design of a spray-dried solid dispersion using RIT as a model API. Therefore, the spray-dried dispersion of PVA 3-82 and RIT with a 30% DL was investigated vs. the crystalline drug and a marketed formulation. For the manufacturing of this product, ritonavir is processed in hot-melt extrusion with PVP-VA as the matrix polymer [25]. In the X-ray powder diffraction, it can be seen that the marketed ritonavir formulation exhibits crystallinity that shows peaks not linked to the API signals, but rather originating from other formulation components (Figure 8a). The prominent RIT peaks at 6.7, 8.2, 18.0, and 21.4 2θ (deg) cannot be found in the respective XRPD of the marketed formulation. The spray-dried dispersion shows a fully amorphous diffractogram. The dissolution test shows a fast onset for both formulations, while the ASD with PVA 3-82 shows a higher and steadier increase of released RIT over a time period of 120 min compared to the marketed formulation (Figure 8b).

### 3.5. ASD with Ketoconazole

Ketoconazole is used in superficial fungal infections and is considered a development of older imidazole fungals such as clotrimazole as it is orally available [26]. Ketoconazole is readily dissolved in acidic media; the challenge with this BSC class II API arrives with the pH shift from the stomach to the intestine as is precipitated with the increase of pH. Due to the pKa of KET, it shows low solubility in pH 6.8.

#### 3.5.1. Spray Drying and Dissolution of Ketoconazole 30% DL in FaSSIF

**Figure 9:**
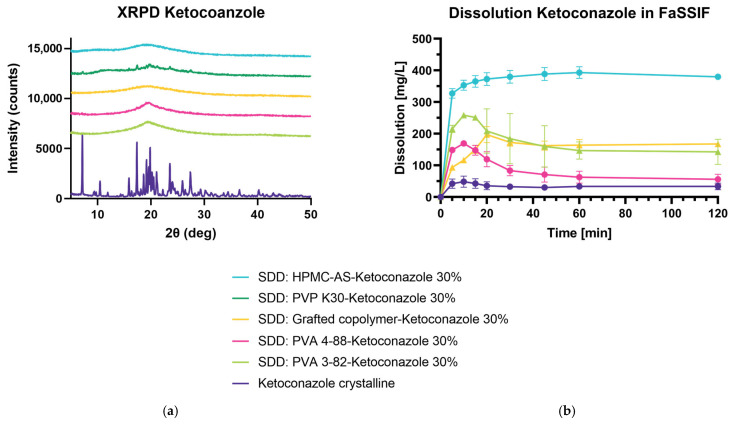
Spray-dried dispersion of 30% DL ketoconazole in FaSSIF. (**a**) X-ray powder diffraction at reflector mode 3°–50° 2θ (deg), scan speed 10° 2θ (deg)min, step size 0.02θ (deg). Acceleration voltage 45 kV and 15 mA current. (**b**) An amount of 400 mg of API was added to 500 mL FaSSIF, Apparatus 2, paddle, 50 rpm at 37 °C. The drug concentration is measured using RP-HPLC.

For this experiment set-up, KET is investigated in FaSSIF with all polymers in a 30% DL. Only the spray-dried dispersion with PVP K30 shows no full conversion to the amorphous state, yielding prominent peaks at 7.1, 17.3, 23.5, and 27.4 2θ (deg) in the diffractogram (Figure 9a). Therefore, it was not included in the dissolution study. For spray drying applications, a PVP with lower viscosity and lower molecular weight might mitigate the challenges we have encountered with IND and KET, where PVP K30 was not able to produce a fully amorphous dispersion. As anticipated, the crystalline drug does not yield high dissolution values in pH 6.8, whereas all other solid dispersions show an enhanced release of KET over time (Figure 9b). HPMC-AS outperforms all other formulations significantly. Followed by the HPMC-AS ASD, both PVA 3-82 and the grafted copolymer increase the release of KET. For the PVA 3-82 sample, a concise spring can be determined during the first 20 min of measurement, slowly decreasing to dissolution values comparable to the grafted copolymer. PVA 4-88 performs in an inferior way to the aforementioned ASDs with KET.

#### 3.5.2. Spray Drying and Dissolution of Ketoconaozole 30% DL in pH Shift

**Figure 10:**
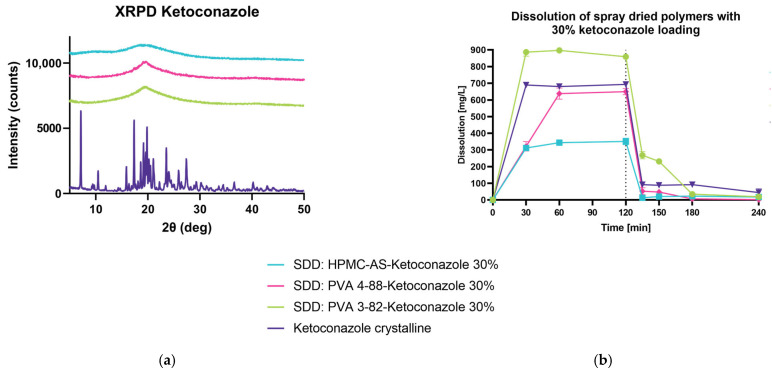
Spray-dried dispersion of 30% DL ketoconazole in pH shift experiments. (**a**) X-ray powder diffraction at reflector mode 3°–50° 2θ (deg), scan speed 10° 2θ (deg)min, step size 0.02θ (deg). Acceleration voltage 45 kV and 15 mA current. (**b**) An amount of 500 mg of KET in 750 mL if 0.1 M HCl, Apparatus 2, paddle, 50 rpm at 37 °C. After 120 min, change of pH from 1.2 to 6.8 with 250 mL 0.2 M Na3PO4*12 H_2_O. Drug concentration is measured using RP-HPLC.

In a second step, we investigated the performance of pH-sensitive polymer HPMC-AS with KET in a 30% DL vs. the PVA 3-82 and PVA 4-88 ASD and the crystalline drug (Figure 10a). Due to the nature of HPMC-AS, only little of the readily dissolved KET can be found in the dissolution media at pH 1.2, whereas PVA 4-88 and the crystalline drug yield similar release values after 60 min, indicating that polyvinyl alcohol 4-88 lets KET freely dissolve (Figure 10b). With the pH shift from pH 1.2 to 6.8 after 120 min, a clear re-crystallization of the API can be determined. While PVA 3-82 supports the highest dissolution values even after the pH shift, HPMC-AS and PVA 4-88 do not outperform the pure crystalline drug.

Comparing the values of free KET after the pH shift experiments and the dissolution performed directly in FaSSIF, PVA 3-82 shows comparable results yielding 258 mg/L after 10 min in FaSSIF vs. 268 mg/L after 10 min in pH 6.8 after the pH shift. Wlodarski et al. investigated the performance of PVA 4-88 in hot-melt extrusion with itraconazole (ITR) in pH shift experiments, coming to the conclusion that this polyvinyl alcohol grade was able to successfully inhibit the precipitation of ITR after the shift from pH 1.2 to pH 6.8 [27].

For the HPMC-AS sample, this consistency cannot be determined, as 350 mg/l of free KET can be found after 10 min in FaSSIF, but almost no free KET is measured 10 min after the shift of pH 1.2 to pH 6.8. In the literature, two influencing factors were identified explaining this behavior. Monschke et al. investigated the dissolution behavior of KET ASDs manufactured by hot-melt extrusion which were milled into different particle sizes using HPMC-AS and Eudragit^®^ L100-55 [28]. Within their work, they describe how phase separation occurs during the exposure of KET in a HPMC-AS matrix to a gastric pH of 1.2, resulting in reduced dissolution rates in the subsequent pH 6.8 measurements. This amorphous–amorphous phase separation (AAPS) was further determined by FT-IR and DSC showing a polymer-rich and API-rich phase when exposed to an acidic pH which can be circumvented using enterically coated capsules. Additionally, Elkhabaz et al. investigated posaconazole with pH-sensitive polymers (HPMC-AS) in pH shift experiments and found that, especially with high-DL ASDs (up to 50% *w*/*w* DL POS in HPMC-AS), re-crystallization of the API occurs within the acidic environment, hindering dissolution in the subsequent pH 6.8 medium [29]. While HPMC-AS was able to generate a stable ASD with posaconazole, it was not able to protect the API from re-crystallization in an acidic environment, and it even protected the crystallized form which led to the crystalline posaconazole transferred into pH 6.8 showing poor dissolution.

## 4. Conclusions

In this paper, we investigated the use of hydrophilic polymers in ASDs manufactured by SD. For this, we used the AMOR–MLKT approach and compared the properties of the generated spray-dried dispersions to crystalline raw material and a formulation generated by a classical two-fluid nozzle set-up using a polymer that was readily co-dissolved with the API in one single liquid feed. In this study, indomethacin, ketoconazole, and ritonavir were used, and we observed that hydrophilic polymers can be used to generate advantageous spray-dried dispersions with all three small molecules. Focusing on a newly developed PVA grade with a hydrolysis degree of 82%, we were able to show conclusive results for the applicability of this polymer in spray-dried dispersions using a three-fluid nozzle experiment design. These findings expand the toolbox of available polymers for formulators, enabling the use of hydrophilic polymers to form an ASD matrix. This could be beneficial considering the release of the final drug product in a gastro-intestinal environment, providing new formulation strategies. The integration of water-based solvents allows the reduction of potential toxic solvents and enables more sustainable manufacturing concepts.

## Figures and Tables

**Table 1:** APIs used in this work and their chemico-physical properties [7,8,9,10,11].

API	Chemical Structure [12,13,14]	BSC	pKa	Solubility at pH 1.2	Solubility at pH 6.8
Indomethacin	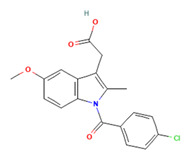	II	4.5	0.0115 mg/mL	0.2843 mg/mL
Ketoconazole	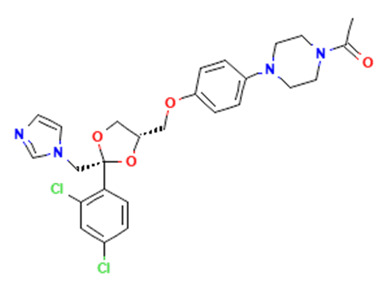	II	2.9 6.5	20.33 mg/mL	0.007 mg/mL
Ritonavir	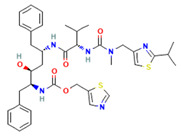	IV	1.8 2.6	0.400 mg/mL	0.001 mg/mL

**Table 2:** Yield in spray drying process of 30% DL ritonavir with used polymers.

Polymer	Yield in SD Process ^1^
PVA 3-82	54%
PVA 4-88	28%
Grafted copolymer	12%
PVP K30	35%
HPMC-AS	1%

^1^ The yield is calculated from the powder obtained in the product vessel in relation to the weighed-in mass for the preparation.

## Data Availability

Data sharing is not applicable to this article.
